# Case Report: Cerebrotendinous xanthomatosis

**DOI:** 10.4103/0971-3026.57218

**Published:** 2009-11

**Authors:** Amit A Karandikar, S Pushparajan, Madhavan N Unni, R Srinivas

**Affiliations:** Department of Radiology, Kerala Institute of Medical Sciences, Trivandrum, Kerala, India

**Keywords:** Cerebrotendinous xanthomatosis, cholestanol

## Abstract

Cerebrotendinous xanthomatosis is a rare genetic disorder. We present and discuss the clinical, radiological, and histopathologic findings in a 36-year-old woman who had juvenile cataract, childhood diarrhea, mental retardation, cerebellar ataxia, and bilateral Achilles tendon xanthomas. She was thoroughly investigated radiologically and biopsy confirmed the diagnosis of xanthomas.

## Introduction

Cerebrotendinous xanthomatosis (CTX) is a rare autosomal recessive lipid storage disorder characterized by accumulation of cholesterol and cholestanol in various tissues, predominantly the central nervous system (CNS), tendons, lungs, liver, and kidneys. Juvenile cataract, childhood diarrhea, mental retardation, and cerebellar ataxia, along with tendon xanthomas, are the most prominent features of this disease. Early diagnosis is extremely important as patients benefit from therapy with chenodeoxycholic acid and further progression of the disease can be prevented. We describe the USG, CT scan, and MRI findings in a 36-year-old woman who presented to us with the typical features of the disease.

## Case report

A 36-year-old woman presented with difficulty in walking and bilateral posterior lower leg nodules. She had been operated in the childhood for bilateral cataracts. She had a history of severe attacks of diarrhea in childhood. She also had a history of poor scholastic performance. On examination, firm masses were noted on the posterior aspect of the legs above the calcaneum. Neurological evaluation revealed the woman to be mentally retarded. She had cerebellar signs like intention tremors, nystagmus and ataxic gait. A provisional diagnosis of CTX was made.

**Figure 1:**
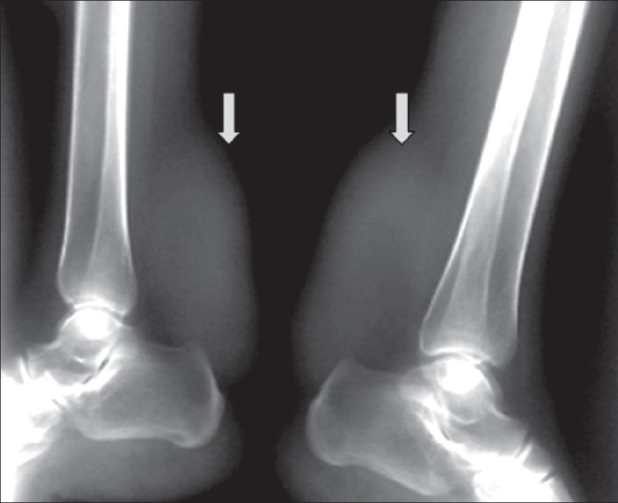
Plain lateral radiograph of both ankles showing bilateral symmetrical swellings (arrows) in the posterior aspect of the lower legs, superior to the calcaneum

**Figure 2 (A-C):**
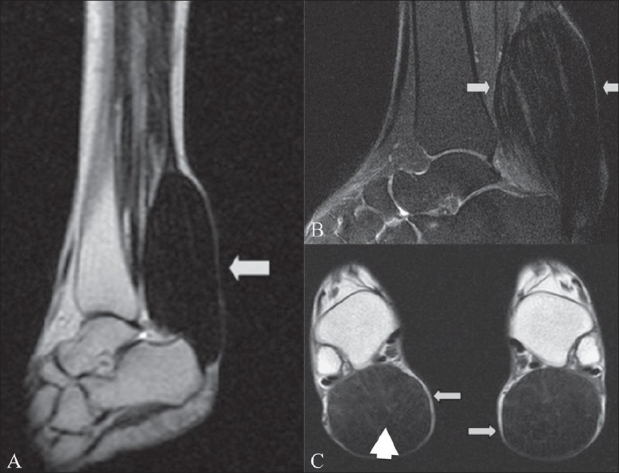
T2W sagittal (A), proton density fat-suppressed sagittal (B) and T1W axial (C) MRI images of the ankles and Achilles tendons show enlarged bilateral Achilles tendons showing classic hypointensities (arrows). A few linear isointense to hyperintense linear lesions are seen within (arrowheads), giving rise to a slightly reticular appearance

Blood investigations revealed a normal complete hemogram and normal levels of serum cholesterol, triglycerides, and all other lipoproteins. Blood cholestanol levels could not be obtained due to non-availability of this test. Plain lateral radiographs of the ankles revealed bilateral soft tissue opacities in the posterior aspect of the lower legs, superior to the calcaneum [Figure 1], without calcification. MRI of both ankles revealed bilateral enlarged Achilles tendons showing hypointensities on T1W and T2W images and proton density (PD) fat-suppressed images. A few linear isointense to hyperintense areas were seen within, giving rise to a slightly reticular appearance [Figure 2].

**Figure 3:**
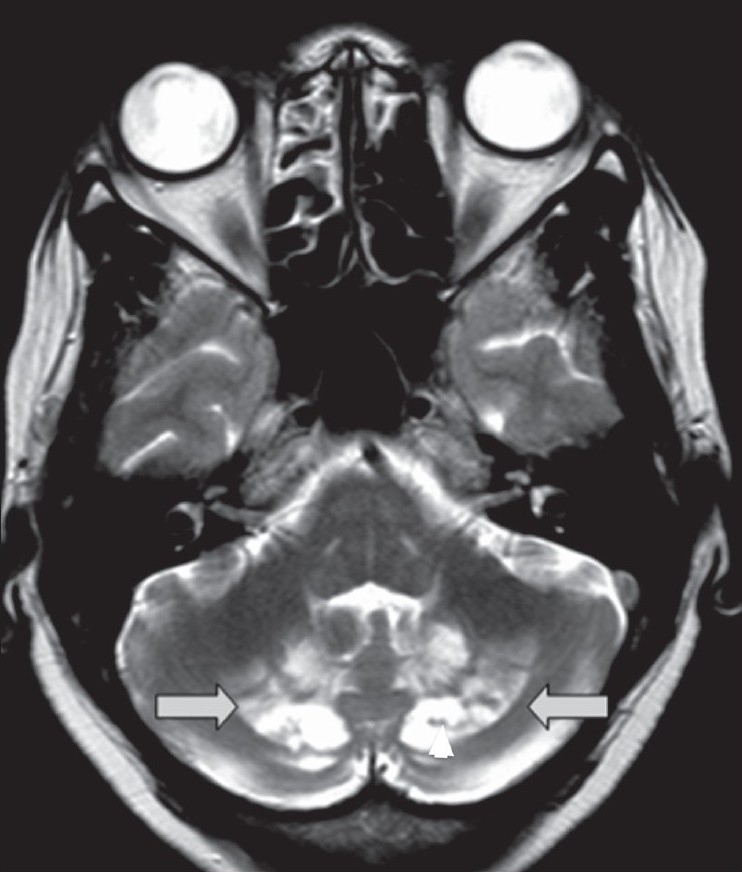
T2W axial MRI image of the brain shows bilateral symmetrical hyperintensities (arrows) involving the dentate nuclei and the deep cerebellar white matter. A few hypointense foci (arrowheads) are seen within the hyperintensities

In view of the neurological signs and symptoms, a brain MRI was also performed. T2W images showed bilateral symmetrical hyperintensities involving the dentate nuclei and the deep cerebellar white matter. The basal ganglia and thalamus were normal. The supratentorial parenchyma was unremarkable [Figure 3].

**Figure 4:**
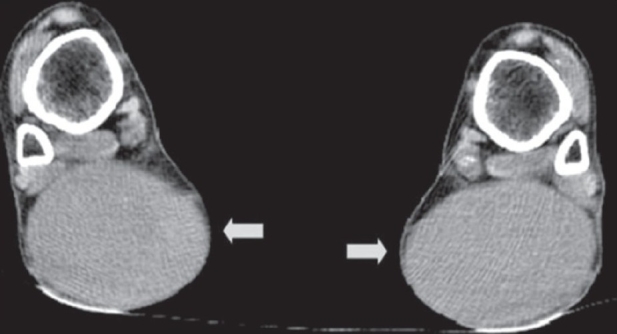
Plain computed tomography scan of both ankles reveals bilaterally symmetric soft tissue density nodules (arrows) in the Achilles tendons. No calcification, hemorrhage, or fat density is seen

**Figure 5:**
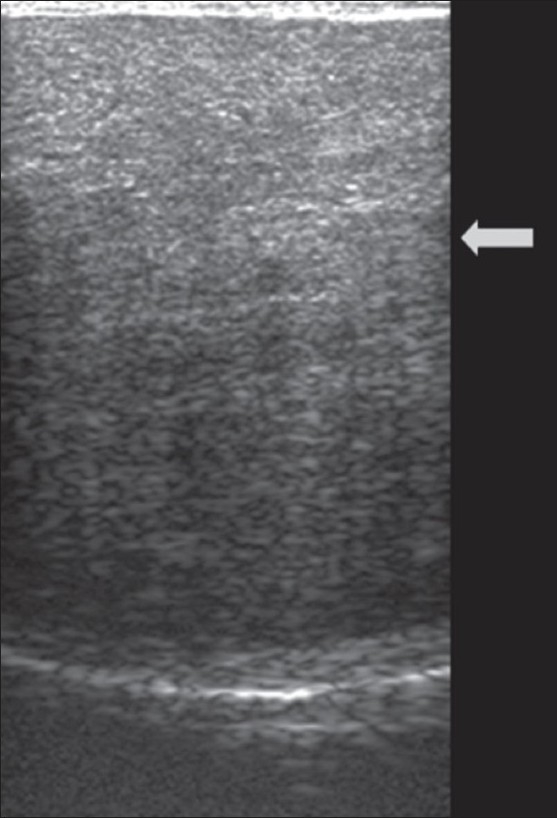
USG of the right Achilles tendon reveals thickening and smooth symmetric hypoechoic infiltration (arrow)

A plain CT scan of the ankles showed soft tissue density lesions in the Achilles tendon, without calcification, fat density, or hemorrhage [Figure 4]. USG revealed loss of the normal fibrillary architecture of both Achilles tendons with thickening and smooth, symmetric, hypoechoic infiltration [Figure 5].

**Figure 6:**
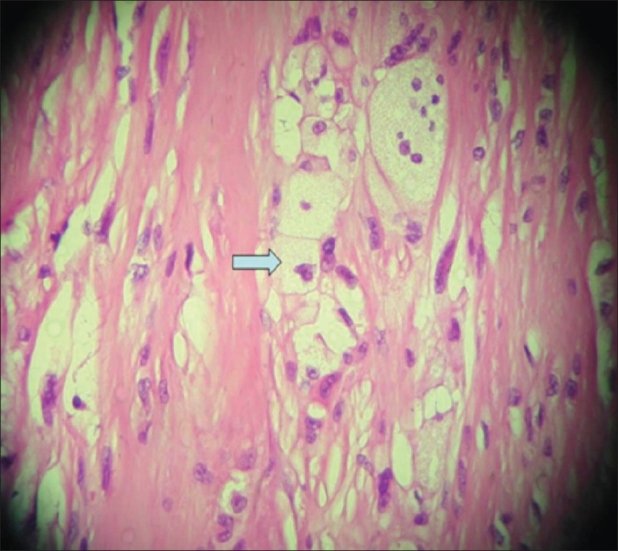
Biopsy of the lesion shows sheets of fibroblasts with collagination and admixed clusters of cholesterol crystals with some areas showing foamy macrophages and multinucleated histiocytic giant cells (arrow). The histopathological appearance is consistent with a xanthoma

A biopsy of the ankle lesions revealed multiple foam cells with eccentric nuclei admixed with fibrotic areas [Figure 6], suggestive of xanthomas.

In view of the typical clinical and radiological features, a diagnosis of CTX was made. She was treated with chenodeoxycholic acid and HMG-CoA reductase inhibitors, after which she showed a mild improvement in her gait with a slight reduction in her Achilles tendon nodules. No change was noted in the mental status.

## Discussion

CTX is a rare autosomal recessive condition caused by a deficiency of the mitochondrial enzyme sterol 27 hydroxylase, which normally catalyses the oxidation of cholesterol to bile acids. Its absence results in an accumulation of cholesterol and cholestanol in all tissues, giving rise to tendon xanthomas.[1] Molecular genetic analysis has revealed that this disease is associated with a mutation of the CYP 27 gene.[2]

These patients present with diarrhea, cataracts, and psychomotor retardation (in infancy/childhood) followed by development of xanthomas after the second decade.[3] In addition to the Achilles tendons, the quadriceps, triceps, and finger extensor tendons may also be involved. These patients also often present with cerebellar ataxia, spinal cord paresis, and peripheral neuropathy. Our patient had most of these symptoms, i.e. childhood diarrhea, cataracts, mental retardation, cerebellar ataxia, and Achilles tendon xanthomas. Although blood cholesterol levels are normal in patients with CTX, the blood cholestanol levels are characteristically elevated.[1,4]

T2W and FLAIR images reveal bilateral symmetrical hyperintensities involving the dentate nuclei and the deep cerebellar white matter. The basal ganglia and thalami may also be involved. A few hypointense foci are sometimes seen within the hyperintensities and are presumed to be due to hemorrhage/calcification.[5–8] Achilles tendon xanthomas are classically hypointense on T1W and T2W images due to the deposition of free cholesterol and cholestanol rather than triglycerides and fatty acids (which are responsible for the normal hyperintense fat signal on T1W images). The MRI may reveal a reticular/speckled appearance due to interspersed areas of slightly high signal intensity, which are presumably due to secondary edema/inflammation.[9] Certain authors believe that high-resolution USG is as sensitive as MRI in depicting the number and extent of intratendinous lesions. CT scan and USG have been used to monitor the response to treatment as both are equally good in assessing the anteroposterior diameter of the tendons, the reduction of which is a parameter for measuring the success of treatment.[9] However we prefer USG for the same due to lack of radiation and equal efficiency.

Diagnosis is based on the typical clinical history and findings, the presence of normal or low cholesterol in association with raised cholestanol levels, and the characteristic MRI appearance. Biopsy is seldom necessary in the presence of all of these features. Clinically, CTX resembles the Marinesco-Sjogren syndrome, an autosomal recessive disorder characterized by the triad of cerebellar ataxia, congenital cataract, and mental retardation.@ The presence of tendon xanthomas helps differentiate CTX from this condition. This differentiation is important as CTX is a treatable condition.[4]

Conservative management with chenodeoxycholic acid and HMG-CoA reductase inhibitors must be started in these patients.[5] Clinical improvement takes time and the patient must be advised long-term follow-up and periodic evaluation.
